# Erianin suppresses prostate cancer progression and attenuates STAT3 signaling

**DOI:** 10.3389/fonc.2026.1975408

**Published:** 2026-10-21

**Authors:** Zhouli Yue, Yufang Zhao, Jiahui Wang, Feifei Wang, Menglin Wang, Zibo Li, Yucheng Li, Min Li

**Affiliations:** 1 The Fifth Clinical Medical College, Henan University of Chinese Medicine, Zhengzhou, China; 2 College of Pharmacy, Henan University of Chinese Medicine, Zhengzhou, China; 3 Collaborative Innovation Center of Research and Development on the Whole Industry Chain for Yu-Yao in Henan Province, Henan University of Chinese Medicine, Zhengzhou, China; 4 Academy of Chinese Medical Sciences, Henan University of Chinese Medicine, Zhengzhou, China

**Keywords:** erianin, prostate cancer, STAT3, network pharmacology, proliferation

## Abstract

**Background:**

Prostate cancer is one of the most prevalent malignancies in men, but current therapeutic strategies are often limited by suboptimal efficacy and the emergence of drug resistance.

**Methods:**

Cell proliferation, migration, and invasion were assessed using CCK-8, colony formation, wound healing, and Transwell assays in PC-3, 22RV1, and RM-1 cells. Network pharmacology and molecular docking were used to identify candidate targets, and the effects of erianin on target were subsequently examined by western blot analysis. *In vivo* antitumor activity was evaluated in C57BL/6N mice bearing RM-1 tumors. Liver and kidney functions were assessed using biochemical kits, and histopathological changes in major organs were examined by H&E staining.

**Results:**

Among the 48 compounds screened, erianin showed the most potent anti-prostate cancer activity. Further assays showed that erianin reduced colony formation, wound closure, and Transwell invaded-cell counts. *In vivo*, erianin markedly suppressed tumor growth, with the 20 mg/kg dose exhibiting the strongest effect. Network pharmacology and molecular docking predicted STAT3 as a candidate target of erianin, while further analysis indicated that erianin inhibited STAT3 phosphorylation and downregulated its downstream effectors including c-Myc and MMP-9. Safety evaluation revealed that erianin caused only a mild elevation in serum alanine aminotransferase (ALT) without significant histopathological alterations in major organs.

**Conclusions:**

These findings demonstrate that erianin exerts potent anti-prostate cancer activity and that these effects are accompanied by reduced STAT3 signaling. Further functional and target-engagement studies are required to determine whether STAT3 inhibition is necessary for erianin.

## 1 Introduction

According to GLOBOCAN 2022 estimates, prostate cancer ranks as the second most diagnosed malignancy and the fifth leading cause of cancer-related death among men worldwide, with an estimated 1.5 million new cases and 397,000 deaths annually (1, 2). Prostate cancer is predominantly an androgen-dependent tumor, and androgen deprivation therapy remains the standard first-line therapeutic strategy (3). Nevertheless, androgen deprivation therapy is generally accompanied by severe adverse events, including cardiovascular disease, metabolic syndrome, and osteoporosis, seriously impairing patients’ quality of life (4). Currently, conventional therapeutic agents for prostate cancer include docetaxel, abiraterone, and enzalutamide (5, 6). However, frequent drug resistance, obvious toxic side effects, and high treatment costs greatly restrict their long-term clinical application (7, 8). Consequently, the novel and effective therapeutic agents are urgently needed to improve the clinical outcomes of prostate cancer patients.

Signal transducer and activator of transcription 3 (STAT3 Tyr705) is a key transcription factor that regulates cell proliferation, differentiation, and immune responses, playing a crucial role in driving the malignant progression of prostate cancer (9, 10). Constitutive STAT3 activation has been detected in approximately 50% of prostate cancer cases (11). This activation triggers STAT3 nuclear translocation and enhances its transcriptional activity (12, 13), which further upregulates downstream target genes including c-Myc and MMP-9, thereby promoting tumor cell proliferation, invasion, metastasis, and angiogenesis (14–16). Sustained STAT3 activation also contributes to therapeutic resistance in prostate cancer to chemotherapy, targeted therapy, and endocrine therapy. Moreover, elevated STAT3 activity is closely correlated with higher Gleason grade, lymph node metastasis, and poor clinical prognosis (17, 18). Therefore, targeting the STAT3 signaling pathway represents a promising therapeutic strategy for prostate cancer.

Erianin is a low-molecular-weight benzyl compound that possesses multiple biological activities, including antioxidation, antiangiogenesis, and antitumor effects (19–22). Accumulating evidence has revealed that erianin exerts potent suppressive effects on various malignancies, including lung adenocarcinoma, esophageal carcinoma, hepatocellular carcinoma, gastric cancer, colorectal cancer, cervical cancer, and breast cancer (23, 24). Mechanistically, erianin has been reported to suppress tumor progression by inhibiting the STAT3 signaling pathway in hepatocellular carcinoma and esophageal cancer (25, 26). However, the anti-prostate cancer activity of erianin and its potential link to STAT3 regulation remain largely unclear.

In the present study, we identified erianin as a potent anti-prostate cancer agent through screening combined with *in vitro* and *in vivo* functional assays. We further elucidated its underlying mechanism using network pharmacology, molecular docking, and molecular biological validation, and subsequently evaluated its preliminary safety profile in major organs, providing an experimental basis for its future preclinical evaluation of erianin in prostate cancer.

## 2 Materials and methods

### 2.1 Chemicals and reagents

Erianin (WKQ-0000378, 99% purity) was purchased from Lookchem (Chengdu, China). Docetaxel (S29850, 98% purity) was purchased from Yuanye (Shanghai, China). Cell counting kit-8 (CCK-8, KTA1020) was purchased from Abbkine (Wuhan, China). BCA protein assay kit was purchased from Solarbio (Beijing, China). Crystal violet staining solution (G1014) was purchased from Servicebio (Wuhan, China). Ham’s F-12K medium (PM150910) was purchased from Procell (Wuhan, China). RPMI-1640 medium, fetal bovine serum (FBS), and penicillin/streptomycin were purchased from HyClone (Logan, UT, USA). Primary antibodies against p-STAT3 (Tyr705, #9145S) and STAT3 (#30835S) were from Cell Signaling Technology (Danvers, MA, USA). Primary antibodies against c-Myc (67447-1-Ig) and MMP-9 (10375-2-AP) were from Proteintech (Wuhan, China). The β-actin antibody (AC026) and HRP-conjugated goat anti-rabbit IgG secondary antibody (AS014) were obtained from ABclonal (Wuhan, China).

### 2.2 Cell culture

Human prostate cancer PC-3 and 22RV1 cells, and mouse prostate cancer RM-1 cells were obtained from the Cell Bank of the Chinese Academy of Sciences (Shanghai, China). RM-1 and 22RV1 cells were cultured in complete RPMI-1640 medium containing 10% FBS and 1% penicillin-streptomycin. PC-3 cells were cultured in complete Ham’s F-12K medium containing 10% FBS and 1% penicillin-streptomycin in a 5% CO_2_ atmosphere at 37 °C.

### 2.3 Cell viability assay

PC-3 (1×10^4^ cells/well), 22RV1 (1.2×10^4^ cells/well), and RM-1 (5×10^3^ cells/well) cells were seeded into 96-well plates and cultured for 24 h. After being treated with different compounds for 24 hours, the culture medium was replaced with one containing 10% CCK-8 solution. After 1 h of incubation, the absorbance at 450 nm was measured using a microplate reader (BioTek, Synergy LX, VT, USA).

### 2.4 Colony formation assay

Cells (1×10^3^ cells/well) were seeded into 6-well plates. Once small clones formed, they were treated with erianin. Cells were then fixed with 4% paraformaldehyde for 15 min and stained with 0.5% crystal violet solution for 30 min. Colonies containing more than 50 cells were counted under a microscope.

### 2.5 Wound healing migration assay

The wound healing assay was carried out as described previously with minor modifications (27, 28). Briefly, PC-3 (3.0 × 10^5^ cells/well), 22RV1 (5.0 × 10^5^ cells/well), and RM-1 cells (1.0 × 10^5^ cells/well) were cultured in 6-well plates until 90 % confluency. The confluent monolayer cells were carefully scratched using a 200 μL tip, and imaged under a light microscope at 0, 12, 24, or 48 h. The wounded area was calculated according to the formula: (mean wounded breadth - mean remained breadth)/mean wounded breadth × 100% (Zhang et al.2025; Xing et al.2020).

### 2.6 Transwell assay

Matrigel-coated Transwell chambers (BD Biosciences, San Jose, CA, USA) were used to assess invasion-related phenotypes. Cells suspended in serum-free medium were seeded into the upper chambers at 3 × 10^4^ cells per chamber for PC-3, 5 × 10^4^ cells for 22RV1, and 1× 10^4^ cells for RM-1. Medium containing 20% FBS was added to the lower chamber as a chemoattractant. PC-3 and RM-1 cells were allowed to adhere and initiate invasion for 24 h, whereas 22RV1 cells were incubated for 48 h. Erianin was then added to the upper chamber at final concentrations of 0.04, 0.4, or 4 μM, and all cell lines were exposed to erianin for an additional 24 h. At the endpoint, non-invading cells were removed from the upper surface of the filter using a cotton swab, while cells on the lower surface were fixed with methanol and stained with crystal violet. Subsequently, cells that had invaded through the membrane were counted under a microscope in randomly selected high-power fields.

### 2.7 RM-1 prostate tumor mouse model

Thirty male C57BL/6N mice (SPF grade, 6–8 weeks old, weighing 21–25 g) were purchased from Beijing Vital River Laboratory Animal Technology Co., Ltd (Beijing, China), and housed under controlled conditions (22-25 °C, 50 ± 5% humidity, 12-h light/dark cycle) with free access to food and water. All experimental procedures were conducted in accordance with the National Institutes of Health guide for the care and use of Laboratory Animals (NIH Publications No. 8023, revised 1978) and approved by the Committee of Animal Care of the Henan University of Chinese Medicine (DWLLLHJJ202601018).

After one week of acclimatization, RM-1 cells (5×10^5^ cells) were subcutaneously injected to induce tumor formation. Mice were randomly divided into five groups (n=6 per group): control, erianin (10, 20, and 40 mg/kg), and docetaxel (10 mg/kg) groups. Erianin was administered by daily intraperitoneal injection (29, 30), and docetaxel was administered via intraperitoneal injection once weekly (31). The control group received an equal volume of saline. Tumor volume was measured every day using a digital caliper and calculated as (length × width^2^ × 0.5). On day 17 post-treatment, mice were euthanized with tribromoethanol (250 mg/kg, i.p.), and tumor tissues were excised, weighed, and processed for formalin-fixed paraffin-embedding or snap-freezing in liquid nitrogen and stored at -80 °C. Whole-blood samples were collected from the retro-orbital venous plexus and divided into two aliquots. One aliquot was collected into EDTA-containing tubes and used for hematological parameters analysis. The remaining blood was centrifuged to isolate serum for biochemical analysis.

### 2.8 Western blot analysis

Total proteins were extracted from cells or tumor tissues using RIPA lysis buffer supplemented with protease/phosphatase inhibitors (CWBIO, Beijing, China). Protein concentration was detected using the BCA assay. The protein samples (10 μg) were separated by SDS-PAGE and transferred to PVDF membrane (IPVH00010, Millipore, Shanghai, China). The membranes were blocked with 5% skim milk for 2 h and incubated overnight at 4°C with primary antibodies against p-STAT3 (Tyr705) (1:1000), STAT3 (1:1000), β-actin (1:10000), c-Myc (1:1000), MMP-9 (1:1000). After washing three times with PBST, the membranes were incubated with goat anti-rabbit secondary antibody (1:10000) for 50 min at room temperature. Protein bands were visualized using ECL reagent (WBKLS0500, Merck, Darmstadt, Germany) and quantified using ImageJ software v1.8.0 (National Institutes of Health, Bethesda, MD, USA). β-actin was used as an internal control.

### 2.9 Network pharmacology analysis

The SwissTargetPrediction, Comparative Toxicogenomics Database, and GeneCards databases were used to predict potential targets for erianin. The GSE55945 dataset (containing 8 normal cases and 13 disease cases) and GSE200879 dataset (containing 9 normal cases and 128 disease cases) was downloaded from the GEO database. The GEO2R platform was used to screen for differentially expressed genes (DEGs). Differential expression analysis was conducted independently for each dataset and volcano plots were generated separately. Subsequently, the differentially expressed genes from the two datasets were intersected with the predicted targets of erianin, obtain potential candidate targets. Both volcano plots and Venn diagrams were generated using OmicStudio (Lianchuan Bio Cloud Platform, Hangzhou, China). For the volcano plot, DEGs were identified based on an absolute fold change > 1.2 and a *P*-value < 0.05. Protein-protein interaction (PPI) networks were constructed using the STRING database (with a confidence score > 0.15) and visualized using Cytoscape software v3.10.0. Core targets were subsequently identified based on node degree centrality. Kyoto Encyclopedia of Genes and Genomes (KEGG) pathway enrichment analyses were performed using DAVID and the Bioinformatics online platform.

### 2.10 Molecular docking

Molecular docking was performed using AutoDock Tools v1.5.7 and PyMOL software. The 3D structure of erianin (CID: 356759) was obtained from PubChem, and the crystal structure of STAT3 (PDB ID: 6DLG) was downloaded from the Protein Data Bank (PDB). Water molecules and native ligands were removed from the STAT3 crystal structure prior to docking. We adopted a full-protein grid coverage strategy with grid dimensions of 58.0 Å × 72.0 Å × 90.0 Å to unbiasedly search all potential binding sites of erianin on STAT3 and avoid bias from predefined binding pockets. Among all predicted conformations, the pose with the lowest binding energy (−7.1 kcal/mol) localized to the SH2 domain adjacent to the Tyr705 phosphorylation site of STAT3 and formed key hydrophobic interactions with LEU-618.

### 2.11 Blood biochemical analysis

The serum levels of blood urea nitrogen (BUN), creatinine (CRE), aspartate aminotransferase (AST), and alanine aminotransferase (ALT) were measured according to the instructions of the kit (Jiancheng Bioengineering Institute, Nanjing, China). The hematological parameters were detected using three-part differential blood cell analyzer (BC-2800vet, Mindray, Shenzhen, China).

### 2.12 Hematoxylin and eosin staining

Tumor and organ tissues were fixed in 4% paraformaldehyde for 24 h, dehydrated, embedded in paraffin, and sectioned at 5-μm thickness. The sections were then stained with hematoxylin and eosin, and examined under a light microscope (Eclipse E100, Nikon, Tokyo, Japan).

### 2.13 Statistical analysis

Data are presented as mean ± SEM and statistical analyses were performed using SPSS 27.0 software (IBM, Armonk, NY, USA). For endpoint comparisons involving multiple groups, data were analyzed using one-way analysis of variance (ANOVA) followed by Tukey’s *post hoc* test. Data distribution was assessed using the Shapiro–Wilk test. Serial estimates of tumor volume are presented descriptively as mean ± SEM and were not subjected to inferential statistical analysis. A value of P < 0.05 was considered statistically significant.

## 3 Results

### 3.1 Activity screening of anti-prostate cancer compounds

To identify compounds with potential anti-prostate cancer activity, we screened 48 compounds from an internally curated exploratory antitumor compound collection established in our laboratory. All compounds were tested at 10 μM for 24 h in PC-3, 22RV1, and RM-1 cells under the same experimental conditions (**Supplementary Table S1**). The compounds were ranked according to their mean inhibitory effects across the three cell lines. The four highest-ranked compounds were erianin (D6), schisandrin (B6), cryptotanshinone (B1), and matrine (B5), which were subsequently selected for concentration-response and IC_50_ analyses (**Figures 1A–D**). Further, using docetaxel as a positive control, we constructed concentration-effect curves for these four compounds and determined their half-maximal inhibitory concentration (IC_50_) values. The results confirmed that erianin showed the most significant anti-proliferative activity in all three cell lines (**Figures 1E–G**), with IC_50_ values of 0.592, 1.985, and 0.442 μM for PC-3, 22RV1, and RM-1 cells, respectively (**Figure 1H**).

**Figure 1:**
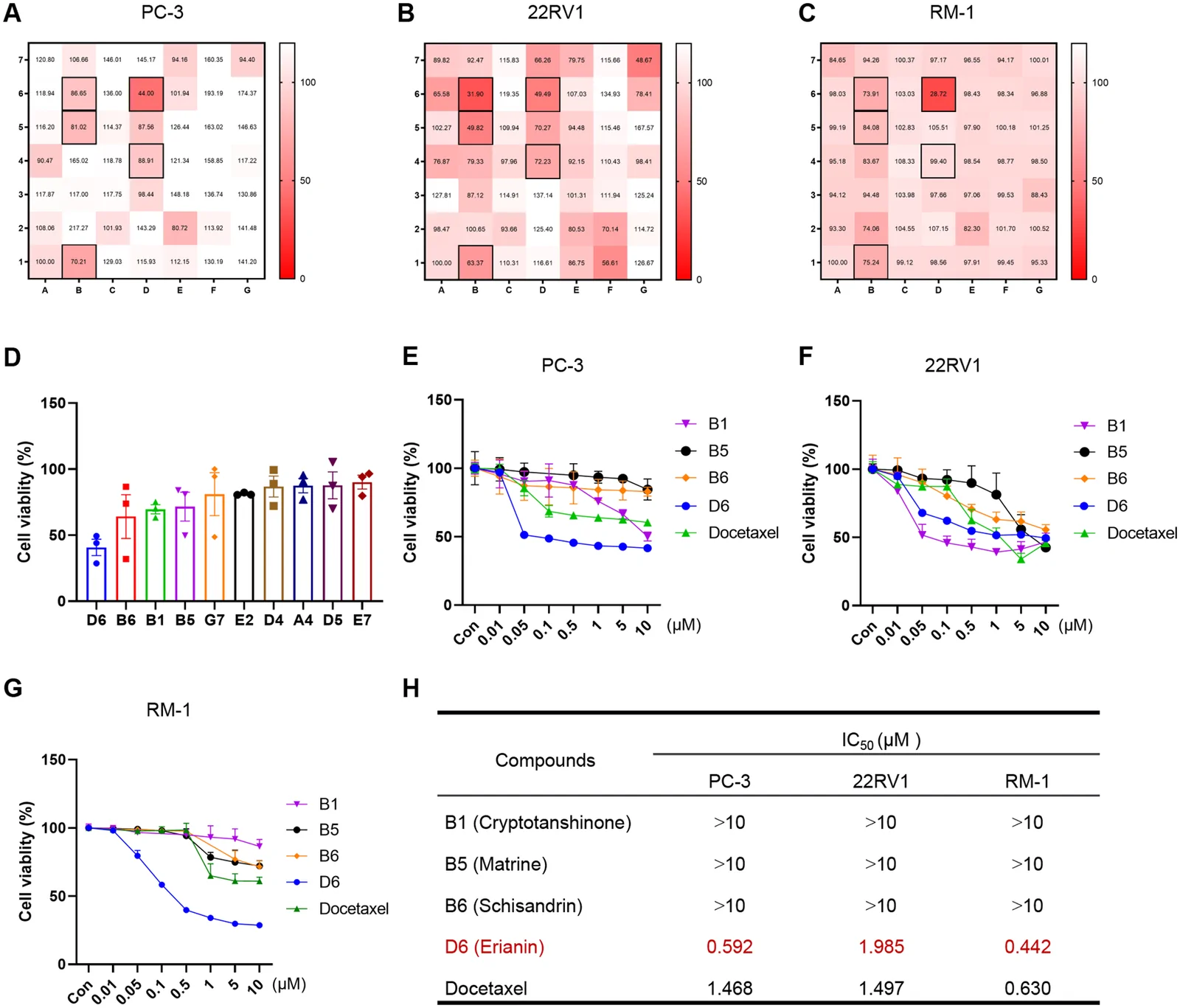
Comparison of the anti-prostate cancer activities of different compounds. **(A–C)** Heat map showing the anti-prostate cancer activity of 48 compounds in PC-3, 22RV1, and RM-1 cells (n = 3). **(D)** The top ten ranked compounds in terms of average inhibition rate: PC-3, 22RV1, and RM-1. **(E–G)** The concentration-response curves of anti-prostate cancer activity (n = 6). **(H)** IC_50_ values of the tested compounds. All data are presented as mean ± SEM and analyzed by one-way ANOVA followed by Tukey’s *post hoc* test.

### 3.2 Erianin suppresses the proliferation and the migratory/invasive phenotype of prostate cancer cells *in vitro*

Cells were treated with three concentrations of erianin (0.04, 0.4, and 4 μM) and evaluated for cell proliferation, as well as migration- and invasion-related phenotypes. The results showed that erianin significantly inhibited colony formation in a concentration-dependent manner (**Figures 2A, B**). Additionally, the wound healing rate was significantly reduced by erianin (**Figures 2C, D**), and the number of invasion cells also decreased significantly with increasing erianin concentration (**Figures 2E, F**), These results indicate that erianin can effectively inhibit the proliferation and the migratory/invasive phenotype of prostate cancer cells *in vitro*.

**Figure 2:**
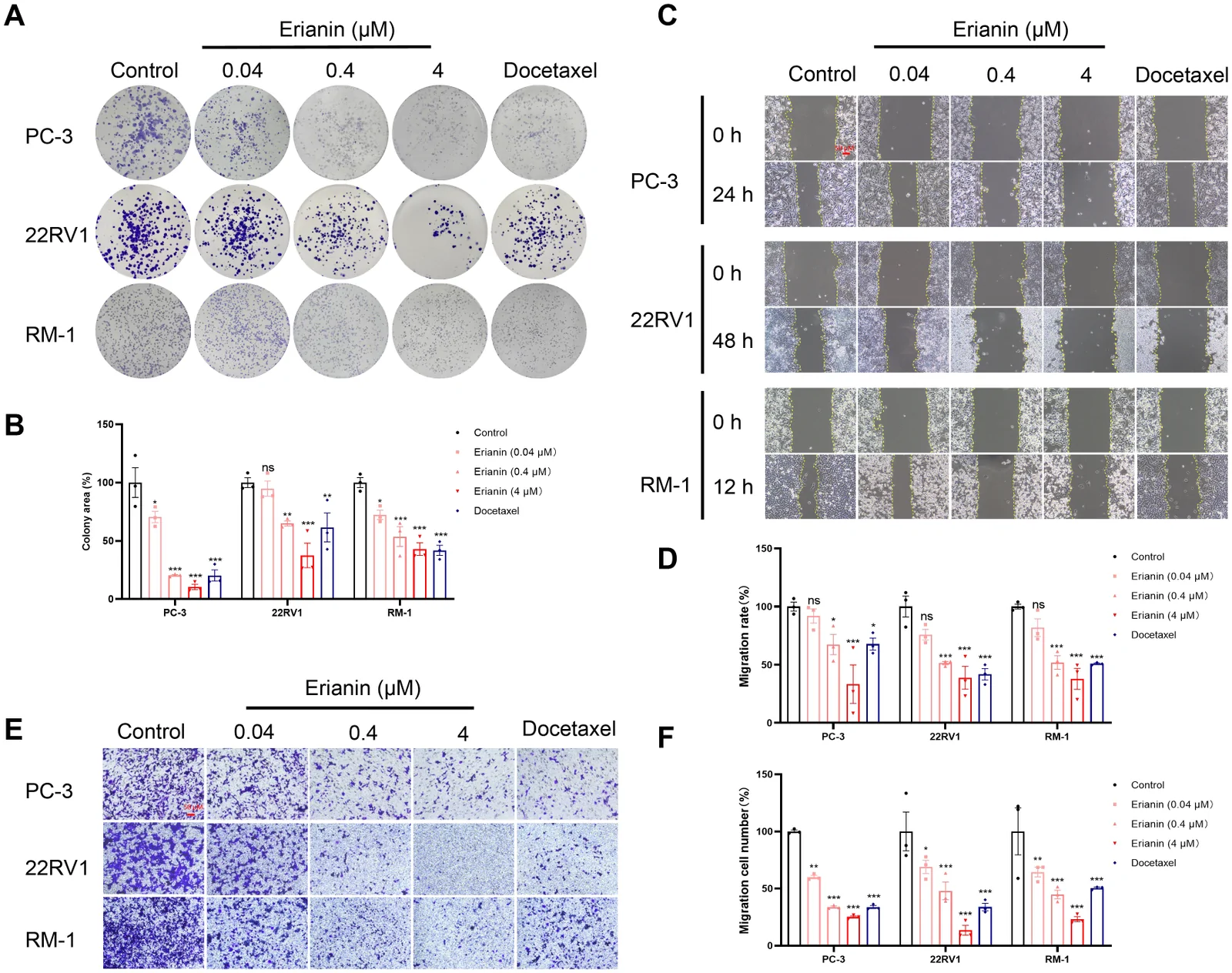
Erianin suppresses the proliferation and the migratory/invasive phenotype of prostate cancer cells *in vitro* **(A)** Representative image of colony formation assays in PC-3, 22RV1, and RM-1 cells (n = 3). **(B)** Quantitative analysis of colony formation efficiency. **(C)** Representative image of the wound healing assay in three cell lines (n = 3). **(D)** Quantitative analysis of wound closure. **(E)** Representative image of Transwell assay in three cell lines (n = 3). **(F)** Quantitative analysis of invaded-cell counts. All data are presented as mean ± SEM and analyzed by one-way ANOVA followed by Tukey’s *post hoc* test. *P < 0.05, **P < 0.01, and ***P < 0.001 vs. control group. Scale bar: 50 μm. ns, no significance.

### 3.3 Screening of targets for the anti-prostate cancer effect of erianin

To explore the underlying mechanisms of erianin’s anti-prostate cancer effects, we generated a volcano plot to visualize DEGs derived from the integrated GSE55945 and GSE200879 datasets (**Figures 3A, B**). A total of 27 potential targets shared by erianin and prostate cancer were identified (**Figure 3C**). KEGG enrichment analysis revealed that these genes were enriched in multiple pathways associated with cell proliferation and adhesion, including EGFR tyrosine kinase inhibitor resistance, PD-L1 expression and PD-1 checkpoint pathway in cancer, and pathways in cancer, suggesting its central role in the antitumor mechanism of erianin (**Figure 3D**). PPI analysis revealed that STAT3 had the highest degree value in the network (**Figures 3E, F**). Molecular docking predicted a possible interaction pose between erianin and the SH2 domain of STAT3, with a calculated binding energy of −7.1 kcal/mol and a predicted hydrophobic interaction involving LEU-618. (**Figure 3G**). These results indicate a possible interaction between erianin and STAT3.

**Figure 3:**
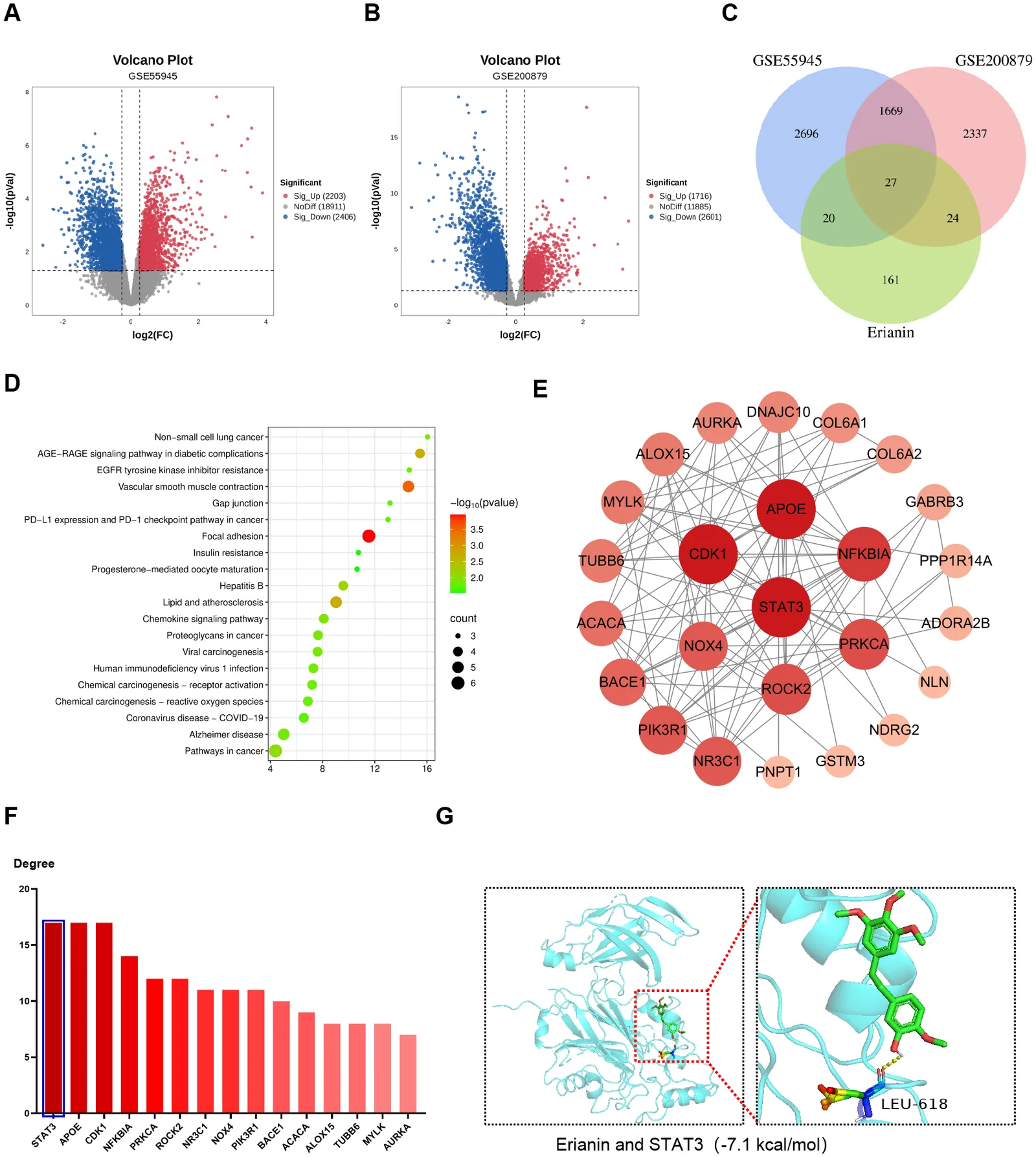
Network pharmacology-based screening of targets underlying the anti-prostate cancer effects of erianin. **(A, B)** Volcano plot showing differentially expressed genes in the GSE55945 and GSE200879 datasets. **(C)** Venn diagram illustrating the overlapping targets between erianin and prostate cancer. **(D)** KEGG enrichment analysis of the 27 overlapping targets. **(E)** PPI network interaction analysis. **(F)** Top 15 hub genes ranked by degree value. **(G)** Molecular docking analysis of erianin with STAT3. KEGG, Kyoto Encyclopedia of Genes and Genomes; PPI, Protein-protein interaction.

### 3.4 Erianin inhibits the phosphorylation of STAT3 *in vitro*

To determine whether erianin affects STAT3 activation, we assessed the protein levels of STAT3 and p-STAT3 (Tyr705) in three cell lines treated with erianin. The results showed that p-STAT3 (Tyr705) protein was significantly downregulated by erianin in all three cell lines, while total STAT3 protein did not show any significant change. In addition, the protein levels of c-Myc were significantly decreased in both PC-3 and RM-1 cells, while in 22RV1 cells, c-Myc protein also showed a decreasing trend, although without statistical significance. At the same time, the protein expression of MMP-9 in all three cell lines showed significant downregulation by erianin (**Figures 4A–F**). These results demonstrated that erianin may inhibit the phosphorylation of STAT3, thereby downregulating its downstream target proteins including c-Myc and MMP-9.

**Figure 4:**
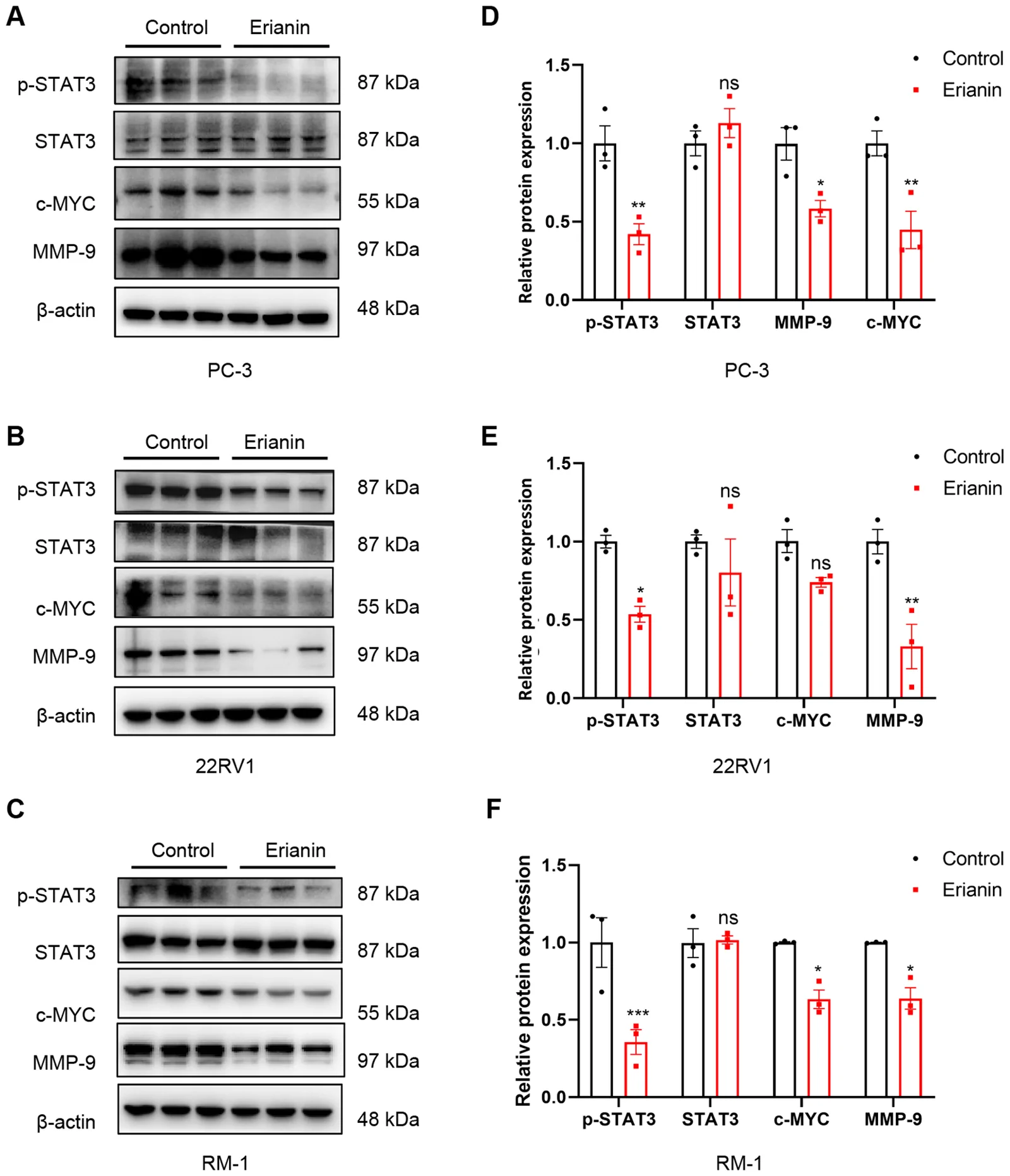
Erianin downregulates the protein expression of p-STAT3, c-Myc, and MMP-9 in prostate cancer cells. **(A–C)** Western blot analysis of proteins expression in PC-3, 22RV1, and RM-1 cells, respectively. **(D–F)** Quantitative analysis of protein expression in the corresponding cell lines (n = 3). All data are presented as mean ± SEM and analyzed by one-way ANOVA followed by Tukey’s *post hoc* test. *P < 0.05, **P < 0.01, and ***P < 0.001 vs. control group. ns, no significance.

### 3.5 *In vivo* verification of the anti-prostate cancer activity of erianin

To further verify the anti-prostate cancer activity of erianin *in vivo*, male mice were subcutaneously injected with RM-1 cells to establish a tumor transplantation model. The descriptive tumor-growth curves showed lower mean estimated tumor volumes in the erianin-treated groups during the treatment period (**Figure 5A**). At the experimental endpoint, tumor weights were significantly lower in the erianin-treated groups than in the control group. Among the treatment groups, the 20 mg/kg dose group exhibited the most significant antitumor effect (**Figures 5A–C**). There was no significant difference in body weight among the groups (**Figure 5D**). In addition, erianin treatment significantly downregulated the protein expression levels of p-STAT3 (Tyr705), downstream effect protein c-Myc, and MMP-9 in tumor tissues (**Figures 5E, F**). These results further demonstrated *in vivo* that erianin has anti-prostate cancer activity and is associated with reduced STAT3 signaling.

**Figure 5:**
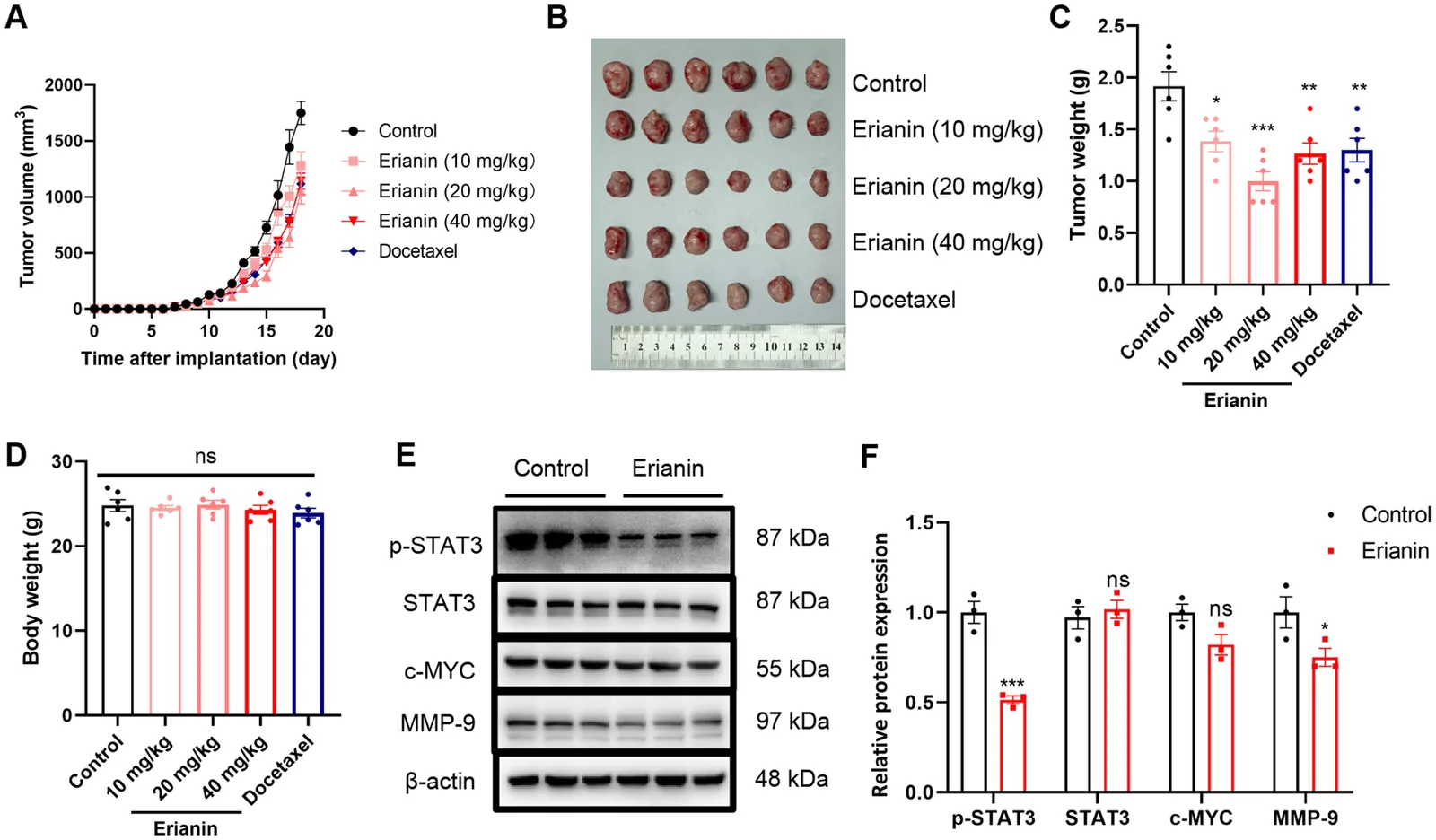
Erianin inhibits tumor growth in tumor-bearing mice. **(A)** Tumor growth curves (n = 6). **(B)** The image of harvested tumors (n = 6). **(C)** Tumor weights (n = 6). **(D)** Body weight of mice (n = 6). **(E)** Western blot analysis of proteins expression *in vivo* (n = 3). **(F)** Quantitative analysis of protein expression. Tumor-volume trajectories are presented descriptively as mean ± SEM without inferential statistical testing. Endpoint data are presented as mean ± SEM and analyzed using one-way ANOVA followed by Tukey’s *post hoc* test; *P < 0.05 and ***P < 0.001 vs. control group. ns, no significance.

### 3.6 Preliminary evaluation of the *in vivo* safety of erianin

To preliminarily assess the safety of erianin, we conducted gross dissection and H&E staining of the major organs of mice, and measured serum ALT, AST, BUN, and CRE levels to evaluate the impact on liver and kidney functions. The results showed that serum AST, BUN, and CRE levels in each dose group did not show significant abnormalities. Although the ALT activity in the erianin-treated groups showed a slightly increase, the values remained within the normal range for healthy mice (**Figures 6A–D**). H&E staining revealed no obvious pathological changes in the heart, liver, spleen, lungs, or kidneys (**Figure 6E**). In addition, platelet counts were slightly lower in the erianin-treated groups than in the control group but remained within the reference interval (**Supplementary Table S2**). These findings provide preliminary information regarding the tolerability of erianin under the tested experimental conditions.

**Figure 6:**
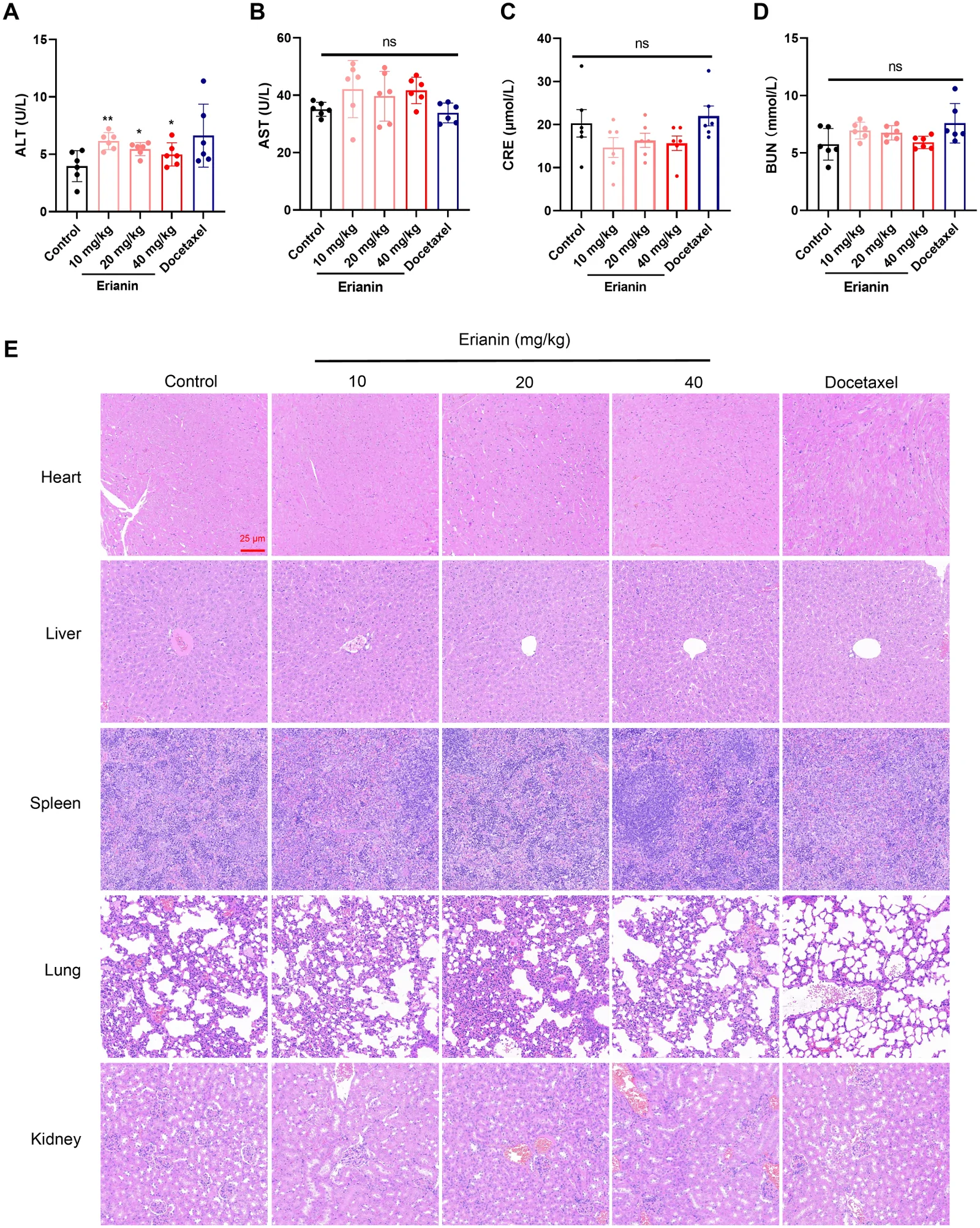
Preliminary evaluation of the *in vivo* safety of erianin. **(A)** Serum ALT activity (n = 6). **(B)** Serum AST activity (n = 6). **(C)** Serum CRE levels (n = 6). **(D)** Serum BUN levels (n = 6). **(E)** Representative H&E staining images of heart, liver, spleen, lung, and kidney (n = 3). All data are presented as mean ± SEM and analyzed by one-way ANOVA followed by Tukey’s *post hoc* test; *P < 0.05 and **P < 0.01 vs. control group. ns: no significance. Scale bar: 25 μm.

## 4 Discussion

Erianin has been demonstrated to exhibit inhibitory effects against various types of cancer (32, 33). However, its activity against prostate cancer and the underlying mechanism remains poorly understood. In the present study, we demonstrated that erianin suppresses prostate cancer progression *in vitro* and *in vivo*, and it may be related to the inhibition of STAT3. To systematically evaluate the anti-prostate cancer potential, our study conducted an activity screening of 48 compounds with reported significant anti-tumor activity. The experiment selected three representative prostate cancer cell lines, including PC-3, 22RV1, and RM-1 cells, to examine the effects of erianin in prostate cancer cell lines with different species origins and androgen receptor-related characteristics. The initial screening results showed that cryptotanshinone, matrine, schisandrin, and erianin all exhibited concentration-dependent inhibition of cell viability. Among the tested compounds, erianin exhibited the most potent *in vitro* anti-proliferative activity. It is worth noting that in the PC-3 and RM-1 cells, erianin exhibited lower IC_50_ values than docetaxel. Furthermore, the results from colony formation and wound healing experiments further confirmed the anti-prostate cancer activity of erianin. To preliminarily explore the potential mechanism associated with the anti-tumor effects of erianin, we performed a network pharmacology analysis by intersecting the differentially expressed genes (DEGs) from GSE55945 and GSE200879 two prostate cancer datasets with the predicted targets of erianin, and STAT3 was identified as a core candidate target among the overlapping genes. STAT3 is a signal transducer and activator of transcription that participates in the process of cell proliferation, differentiation, apoptosis, invasion, and migration (34). In malignant tumors, STAT3 Try705 undergoes phosphorylation and translocates to the nucleus, where it functions as a transcription factor and drives the expression of downstream effect molecules, including MMP-9 and c-Myc, both of which play critical roles in tumor cell proliferation and metastasis (35, 36). Consequently, pharmacological inhibition of STAT3 Try705 ultimately suppresses tumor cell proliferation, migration, and invasion by repressing oncogenic transcription (37, 38). Tyr705 phosphorylation is the essential upstream event driving STAT3 dimerization, nuclear translocation, and transcriptional activation, and has been widely used as a canonical readout of STAT3 activity. Our results demonstrates that erianin can significantly reduce the level of p-STAT3, which is consistent with its observed inhibitory effects on cell proliferation and migration *in vitro*. Previous studies have also confirmed that erianin can inhibit the STAT3 signaling in hepatocellular carcinoma and esophageal cancer (25, 26), further supports that reduced STAT3 signaling may be associated with the observed antitumor effects. However, the present findings do not establish STAT3 as a direct molecular target of erianin. Reduced STAT3 phosphorylation could result from direct interaction with STAT3 or from modulation of upstream kinases, phosphatases, or other signaling regulators. Target-engagement assays and analyses of upstream signaling components will therefore be required to distinguish these possibilities.

To further validate the anti-prostate cancer activity of erianin *in vivo*, we established an RM-1 tumor-bearing mouse model in C57BL/6 mice. Erianin treatment significantly reduced tumor weight across all dose groups, with the 20 mg/kg dose exhibiting the most pronounced antitumor effect, indicating a non-monotonic dose-response under the present experimental conditions. In addition, erianin markedly inhibited STAT3 phosphorylation and reduced MMP-9 expression in tumor tissues, consistent with our *in vitro* findings. Meanwhile, preliminary safety evaluation revealed no obvious toxic reactions in major organs, with only a mild elevation in serum ALT levels observed and the platelet count was slightly lower compared to the normal group, which remained within the normal physiological range. These findings provide preliminary evidence of antitumor activity and tolerability under the tested experimental conditions.

However, several limitations of this study should also be acknowledged. First, this study predominantly establishes a correlative rather than causal relationship between STAT3 signaling inhibition and the anti-tumor effects of erianin. Rescue or loss-of-function experiments are warranted in future research to functionally validate the necessity of STAT3 inhibition. Second, an atypical dose-response relationship was observed in the *in vivo* pharmacodynamic experiments, with the 20 mg/kg dose showing greater tumor suppression than the 40 mg/kg dose. Because pharmacokinetic exposure, formulation stability, and detailed dose-related toxicity were not assessed, the basis of this pattern cannot be determined from the present data. The findings therefore do not establish an optimal dose or a linear dose-efficacy relationship. Additional pharmacokinetic and dose-ranging studies will be required before the *in vivo* dose-response can be interpreted. Third, the three cell lines and the subcutaneous RM-1 model used in this study do not fully recapitulate the molecular, androgen-receptor-related, immune, and clinical heterogeneity of human prostate cancer. Additional studies using androgen-receptor-positive and -negative human prostate cancer models, organoids, or patient-derived models, and clinically relevant metastatic or treatment-resistant models will be required before the translational relevance of erianin can be assessed.

## 5 Conclusion

In conclusion, the present study identifies erianin as a promising anti-prostate cancer compound both *in vitro* and *in vivo*, and suggests its mechanism may be related to the suppression of STAT3 signaling. These findings support further preclinical investigation of erianin in prostate cancer. Validation in more diverse human-derived models, together with pharmacokinetic and comprehensive toxicological studies, is required before its translational potential can be assessed.

## Data Availability

The datasets presented in this study can be found in online repositories. The names of the repository/repositories and accession number(s) can be found in the article/**Supplementary Material**.
