# Supplementary material for: Erianin suppresses prostate cancer progression and attenuates STAT3 signaling
Source: Front Oncol. 2026 Oct 21;16:1975408. doi: 10.3389/fonc.2026.1975408 (PMC13730896; doi:10.3389/fonc.2026.1975408)
Supplement: Supplementary file 1 [file DataSheet1.pdf]

Table S1 Comparison of inhibitory activities of 48 compounds against three prostate cancer cell lines

Number	Compounds	Cell viability (%)			
		PC-3	22RV1	RM-1	Mean
A1	Control	100.00	100.00	100.00	100.00
A2	Ginsenoside Rb2	108.06	98.47	93.30	99.94
A3	Glycyrrhizic acid	117.87	127.81	94.12	113.27
A4	Formononetin	90.47	76.87	95.18	87.51
A5	Tenuigenin	116.20	102.27	99.19	105.89
A6	Presenegenin	118.94	65.58	98.03	94.18
A7	Resveratrol	120.80	89.82	84.65	98.42
B1	Cryptotanshinone	70.21	63.37	75.24	69.61
B2	Luteolin	217.27	100.65	74.06	130.66
B3	Rhein	117.00	87.12	94.48	99.53
B4	Baicalin	165.02	79.33	83.67	109.34
B5	Matrine	81.02	49.82	84.08	71.64
B6	Schisandrin	86.65	31.90	73.91	64.15
B7	Enoxolone	106.66	92.47	94.26	97.80

C1	Quinic acid	129.03	110.31	99.12	112.82
C2	Daidzein	101.93	93.66	104.55	100.05
C3	Atractylenolide III	117.75	114.91	103.98	112.21
C4	Beta-asarone	118.78	97.96	108.33	108.36
C5	Senkyunolide I	114.37	109.94	102.83	109.05
C6	Polygalasaponin F	136.00	119.35	103.03	119.46
C7	Fluoxetine	146.01	115.83	100.37	120.74
D1	Sertraline	115.93	116.61	98.56	110.37
D2	Citalopram	143.29	125.40	107.15	125.28
D3	Duloxetine	98.44	137.14	97.66	111.08
D4	Selegiline	88.91	72.23	99.40	86.85
D5	Venlafaxine	87.56	70.27	105.51	87.78
D6	Erianin	44.00	49.49	28.72	40.74
D7	Mifepristone	145.17	66.26	97.17	102.87
E1	*N*-methylamurensininemethine B	112.15	86.75	97.91	98.94
E2	*N*-methylamurensininemethine C	80.72	80.53	82.30	81.18
E3	*N*-methylamurensininemethine D	148.18	101.31	97.06	115.52

E4	nudicauside A	121.34	92.15	98.54	104.01
E5	(-)-amurine *N*-oxide	126.44	94.48	97.90	106.27
E6	reframine B	101.94	107.03	98.43	102.47
E7	reframidine *N*-oxide B	94.16	79.75	96.55	90.15
F1	*N*-methylamurensininemethine	130.19	56.61	99.45	95.42
F2	reframidine	113.92	70.14	101.70	95.25
F3	reframinne	136.74	111.94	99.53	116.07
F4	amurensinine	158.85	110.43	98.77	122.68
F5	amurensinine *N*-oxide A	163.02	115.46	100.18	126.22
F6	*N*-Methyl-14-*O*-desmethylepiporphyroxine	193.19	134.93	98.34	142.15
F7	Rhoeagenine	160.35	115.66	94.17	123.39
G1	Meconoquintupline	141.20	126.67	95.33	121.07
G2	amurine	141.48	114.72	100.52	118.91
G3	8,14-Dihydroflavinantine	130.86	125.24	88.43	114.84
G4	*N*-methyl-2,3,6-trimethoxymorphinandien-7-one	117.22	98.41	98.50	104.71

G5	amuronine	146.63	167.57	101.25	138.48
G6	Dihydrosanguinarine	174.37	78.41	96.88	116.55
G7	cheilanthifoline	94.40	48.67	100.01	81.03

Table S2 Effects of erianin on hematological parameters in mice

Parameters	Control	Erianin (mg/kg)			Docetaxel
		10	20	40	
WBC (10^9/L)	9.12 ± 1.37	10.52 ± 1.42	11.60 ± 1.00	11.92 ± 0.44	11.90 ± 1.66
Lymph# (10^9/L)	6.73 ± 1.16	6.50 ± 0.99	7.27 ± 0.50	8.02 ± 0.53	8.63 ± 1.45
Mon# (10^9/L)	0.45 ± 0.07	0.55 ± 0.10	0.63 ± 0.09	0.57 ± 0.08	0.60 ± 0.14
Neu# (10^9/L)	1.93 ± 0.27	3.47 ± 0.71	3.70 ± 0.53	3.33 ± 0.61	2.67 ± 0.67
Lymph (%)	72.10 ± 4.35	61.22 ± 4.75	63.23 ± 2.73	67.63 ± 5.24	72.00 ± 7.19
Mon (%)	5.13 ± 0.47	5.58 ± 0.43	5.40 ± 0.33	4.78 ± 0.52	5.33 ± 1.44
Neu (%)	22.77 ± 3.90	33.20 ± 4.35	31.37 ± 2.45	27.58 ± 4.76	22.67 ± 5.80
RBC (10^{12}/L)	7.25 ± 0.20	7.00 ± 0.18	6.76 ± 0.10	6.43 ± 0.08	5.13 ± 0.77
HGB (g/L)	127.17 ± 3.09	120.33 ± 3.03	117.83 ± 1.76	112.83 ± 1.62	115.00 ± 3.01
HCT (%)	34.70 ± 0.93	32.92 ± 0.91	31.95 ± 0.58	29.98 ± 0.41	23.57 ± 3.56
MCV (fL)	47.97 ± 0.46	47.12 ± 0.36	47.35 ± 0.43	46.68 ± 0.33	46.30 ± 1.14
MCH (pg)	17.50 ± 0.09	17.15 ± 0.11	17.35 ± 0.08	17.50 ± 0.07	26.33 ± 5.52
MCHC (g/L)	366.17 ± 3.25	365.33 ± 3.69	368.50 ± 4.87	375.83 ± 3.68	567.17 ± 109.93
RDW (%)	14.60 ± 0.24	14.43 ± 0.21	13.83 ± 0.22	14.42 ± 0.24	25.63 ± 4.50

PLT (10^9/L)	672.83 ± 31.31	640.17 ± 54.29	628.67 ± 38.14**	598.00 ± 28.16***	1771.50 ± 483.41***
MPV (fL)	5.62 ± 0.20	5.62 ± 0.11	5.68 ± 0.22	5.55 ± 0.23	7.18 ± 0.70
PDW	16.55 ± 0.09	16.43 ± 0.11	16.52 ± 0.12	16.50 ± 0.09	17.20 ± 0.51
PCT (%)	0.38 ± 0.02	0.36 ± 0.03	0.36 ± 0.04	0.33 ± 0.02	0.49 ± 0.07

WBC: White blood cell count ; Lymph: Lymphocyte count; Mon: Monocyte count; NEU: Neutrophil count; RBC: Red blood cell count; HGB: Hemoglobin; HCT: Hematocrit; MCV: Mean corpuscular volume; MCH: Mean corpuscular hemoglobin; MCHC: Mean corpuscular hemoglobin concentration; RDW: Red cell distribution width; PLT: Platelet count; MPV: Mean platelet volume; PDW: Platelet distribution width; PCT: Plateletcrit. All data are presented as mean ± SEM and analyzed by one-way ANOVA followed by Tukey's post hoc test; $^{**}P < 0.01$ and $^{***}P < 0.001$ vs. control group. n=6.
